# Bacteriological concentration of peritoneal drainage fluid could make an early diagnosis of anastomotic leakage following rectal resection

**DOI:** 10.1038/s41598-021-02649-6

**Published:** 2021-11-30

**Authors:** Wei Ge, Hai-yan Gong, Yong-quan Xia, Li-hua Shao, Han Shen, Gang Chen

**Affiliations:** 1 Department of General Surgery, Nanjing Drum Tower Hospital, The Affiliated Hospital of Nanjing University Medical School, Nanjing, 210008 Jiangsu People’s Republic of China; 2 Physical Examination Center, Nanjing Drum Tower Hospital, The Affiliated Hospital of Nanjing University Medical School, Nanjing, 210008 Jiangsu People’s Republic of China; 3 Department of Clinical Lab, Nanjing Drum Tower Hospital, The Affiliated Hospital of Nanjing University Medical School, Nanjing, 210008 Jiangsu People’s Republic of China

**Keywords:** Cancer, Biomarkers, Gastroenterology

## Abstract

To investigate that the bacteriological concentration and pH value in peritoneal drainage fluid might serve as indicators of early diagnosis of anastomotic leakage following rectal resection. We prospectively analyzed consecutive patients who were treated for rectal diseases with anastomosis at the department of general surgery, the affiliated hospital of Nanjing University Medical School between August 2018 and December 2020. The bacteriological concentration and the pH value in peritoneal drainage fluid were tested on the first, fourth, seventh days postoperatively. A total of 300 consecutive patients underwent rectal resection were tested. 21 patients present with AL and the overall AL rate was 7%. The bacteriological concentration in peritoneal drainage fluid of AL group was significantly higher than that in non-AL group. The AUC value was 0.98 (95% confidence intervals 0.969–1.000) according to the ROC curve. The best cut-off value was 1143/uL. The sensitivity and specificity were 100% and 93.19% respectively. There was no difference of pH value between the AL and non-AL groups. According the results of present study, a high bacteriological concentration in peritoneal drainage fluid is a good marker for predicting and diagnosing AL following rectal resection. However, owing to the limitation of the sample, there was no validation attempt in the study. A large sample study is needed to validate the conclusion.

## Introduction

Anastomotic leakage (AL) is one of the most serious complications following rectal resection, not only causing general infection and prolonging hospital stay but also promoting tumor recurrence and metastasis^1–4^. So that early diagnosis of AL is very important. Up to now, there was a series of researches focused on exploring the markers for early diagnosis of AL, such as c-reactive protein (CRP), procalcitonin (PCT), leukocyte, cytokine and so on^5,6^. These markers are mainly tested in the serum and the sensitivity and specificity are not very high. Therefore, we should explore new markers to detect AL.

AL is defined as integrity defect of colorectal anastomotic stoma, resulting in communication of internal and external intestinal space by the international study group of rectal cancer (ISREC). According to the definition, the earliest manifestation should be the drainage fluid around the anastomotic site when AL attacks. Therefore, the change of drainage fluid should be the earliest evidence of AL and we should explore markers in peritoneal drainage fluid for early diagnosis of AL. When AL happens, bacteria in the gut can then flow into the abdominal cavity and the concentration of bacteria in the drainage fluid will increase. Therefore, we have a hypothesis that testing bacteriological concentration in peritoneal drainage fluid could diagnose AL. Besides, pathophysiological character of AL is acute inflammation and acidic pH characterizes most inflammatory microenvironments. As a result, when AL present, the peritoneal drainage fluid around the anastomotic stoma is becoming acidic. We image that the pH of drainage fluid may also predict AL. We collected the literature and found few researches focused on this topic.

This prospective study aimed to investigate whether the bacteriological concentration and pH value in peritoneal drainage fluid might serve as indicators of early diagnosis AL following rectal resection.

## Patients and methods

### Patients

Consecutive patients who were treated for rectal diseases with anastomosis at the department of general surgery, the affiliated hospital of Nanjing University Medical School between August 2018 and December 2020 were analyzed prospectively. The inclusion criteria are (1) underwent rectal surgery with anastomosis. Exclusion criteria are (1) acute intestinal obstruction or intestinal perforation, (2) combined with severe cardiopulmonary disease, (3) combined with severe coagulation mechanism disorder. All methods were performed in accordance with the relevant guidelines and regulations and the written informed consents for participation in the study were obtained from all participants. This study was approved by the Institutional Review Board (IRB) of Nanjing Drum Tower Hospital, the affiliated hospital of Nanjing University Medical School.

The preoperative preparations were the same and all the patients underwent cleansing enema and given two boxes of polyethylene glycol electrolyte dispersants. Rectal resection was performed for the screened patients. The anastomosis was made by DST EEA Auto Suture. We took an air test when the anastomosis was completed and repaired the defect when the air test was positive. At the end of the operation, we placed a drainage tube around the anastomosis. We poured the drainage fluid daily until the tube was removed. We collected the age, gender, location of the tumor, surgical approach, stage of cancer, mean postoperative hospital stay and so on. Postoperative complications were also recorded in all patients.

### The test markers

We collected 2 ml drainage fluid from the drainage tube on the first, fourth, and seventh days postoperatively using the sterile drying tube in sterile condition. The specimens were sent to clinical lab immediately to test the concentrations of bacteria and the pH levels. We test the serum CRP, white blood cell (WBC), and PCT on the first, fourth, and seventh days postoperatively synchronize with concentration of bacteria and the pH value. The bacteriological concentration was tested using Fully Automated Urine Particle Analyzer UF-1000i (Sysmex Corporation, Japan.) by flow cytometry technology. The pH value was tested using Fully Automated Urine Analyzer AUTION MAX AX-4030 (ARKRAY Corporation, Japan) by dual wavelength reflection measurement (pH indicator). The reagent of pH test was Urine test strips AUTION Sticks 10EA (ARKRAY Corporation, Japan).

### The patients’ groups

AL was defined clinically as gas, pus, or fecal discharge from the drain, fecal discharge from the operative wound, pelvic abscess, peritonitis, and rectovaginal fistula. The AL should be confirmed by one or more of the following methods: radiological contrast study, CT scan, and digital rectal palpation^7^. The patients present with AL were divided into AL group and the others were divided into non-AL group.

### Statistical analysis

For comparisons, we used two-tailed student *t* test to evaluate the continuous variables. The continuous variables were reported as mean ± standard deviation. *P* < 0.05 was considered statistically significant. All *P* values were two sided. The ROC (receiver operator characteristic) curve was used to determine the best cut-off value. The ROC curves are a plot of the sensitivity (true positives) of the test against 1-specifcity (false positives), for each threshold of the test. Each point on the ROC curve represents a particular pair of sensitivity and (1-specifcity) for each determined threshold. The Confidence Interval is 95%. All statistical calculations were performed using SPSS software (version 19.0).

### Statement of ethics

This study was approved by the IRB of Nanjing Drum Tower Hospital, the affiliated hospital of Nanjing University Medical School.

## Results

### Clinical characteristics of the patients

**Figure 1:**
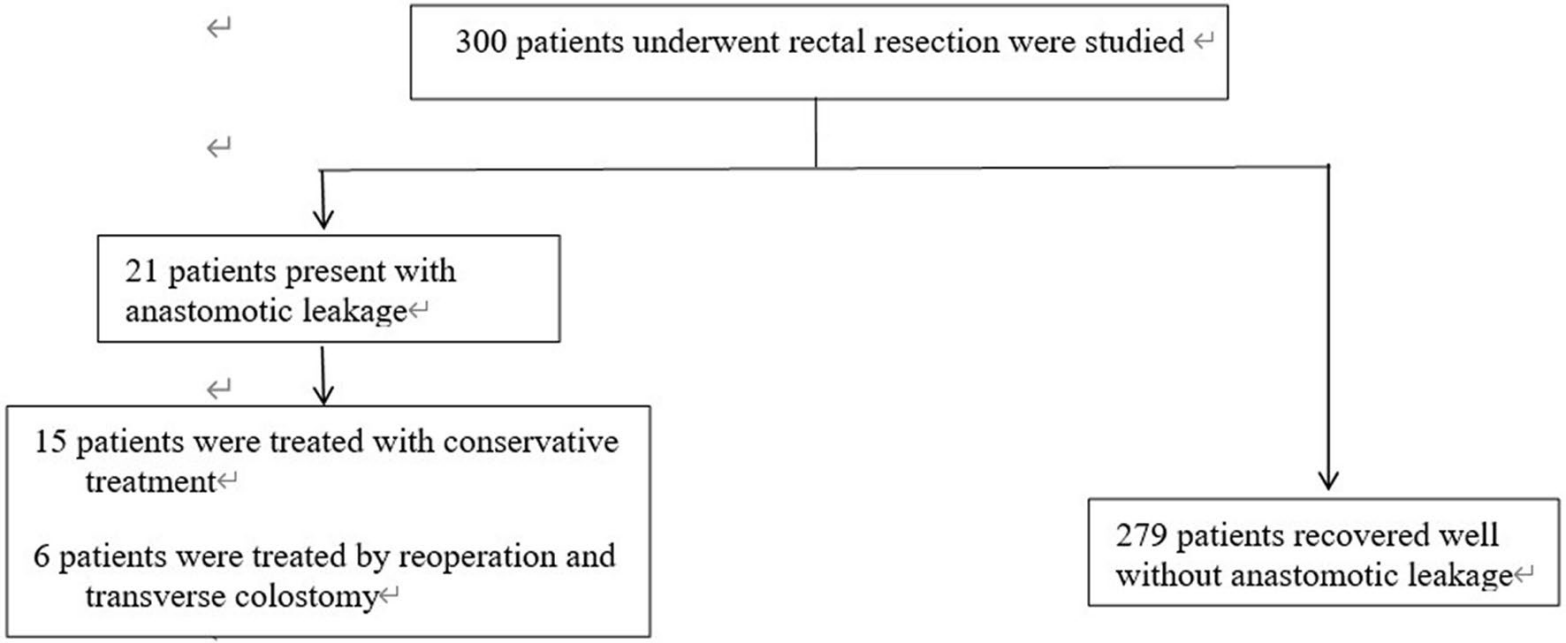
Trial profile.

A total of 300 consecutive patients with rectal disease underwent rectal resection were studied (170 men and 130 women, ages ranging from 23 to 89 years, with mean age 65.8 years). Among these patients, 294 cases underwent laparoscopic surgery and 6 cases underwent open operation. The mean hospital stay after surgery was 8.9 days. According to the diagnostic criteria of AL, there were 21 patients present with AL and the remaining 279 cases recovered well without AL. The overall AL rate was 7%. The details were summarized in Table 1. Of these 21 cases with AL, 12 cases were diagnosed on postoperative day (POD) 6, 7 on POD 7 and 2 on POD 9. Fifteen patients with AL were treated with conservative treatment such as fasting, anti-infection, keeping drainage unobstructed and so on and discharged uneventfully. However, the remaining six patients were treated by reoperation and transverse colostomy (Fig. 1).

**Table 1:** The clinical characteristics of the patients stratified by group.

Variable	Non-AL group	AL group
**Age mean (IQR) years**	63.6 (23–89)	67.9 (46–89)
< 65	155	9
≥ 65	124	12
**Gender**		
Male	158	12
Female	121	9
**Location of tumor (distance from anal margin)**		
< 5 cm	60	10
5-10 cm	122	7
10-15 cm	76	2
> 15 cm	21	2
**Surgical approach**		
Laparotomy	4	2
Laparoscopy	275	19
**Preventive ileostomy**		
Yes	0	0
No	279	21
**Neoadjuvant chemoradiotherapy**		
Yes	70	5
No	209	16
**Operation type**		
Elective operation	269	21
Emergency operation	10	0
**Stage of cancer**		
I	45	2
II	130	13
III	95	6
IV	9	0
Mean postoperative hospital stay (IQR) days	7.3 (5–16)	20.4 (11–50)

### Bacteriological results in drainage fluid

**Figure 2:**
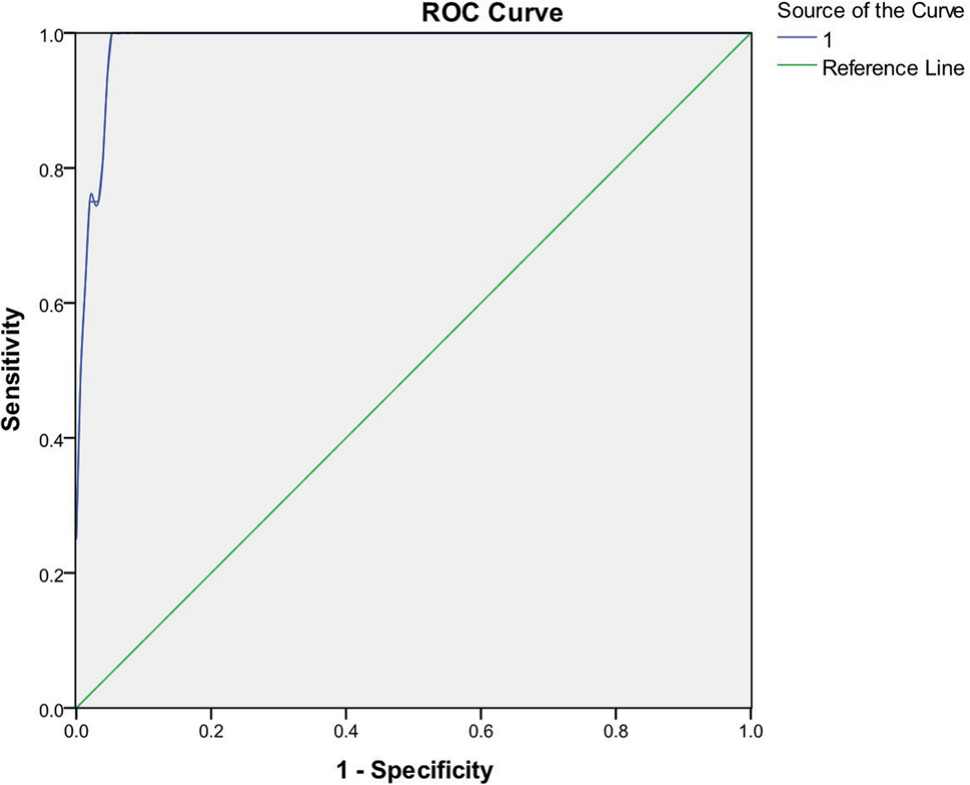
Receiver operator characteristic (ROC) Curve for cut-off analysis of concentration of bacteria value in patients with AL.

We summarized the highest bacteriological concentration in drainage fluid of each patient and compared between the two groups. We found that the highest bacteriological concentrations of 250 patients were on POD 4, and the rest 50 cases were on POD 7. For patients in AL group, we summarized the highest bacteriological concentration in drainage fluid before clinical symptoms. The results showed that the highest bacteriological concentration of each patient in AL group was significantly higher than that in non-AL group (*p* < 0.001) (Table 2). The Receiver Operator Characteristic (ROC) Curve was got in Fig. 2 and the AUC value was 0.98 (95% confidence intervals 0.969–1.000). Based on the evaluation of the ROC curve, a cut-off value of 1143/uL maximized the sensitivity (100%) and specificity (93.19%) of concentration of bacteria value in predicting the risk of AL (Table 3). In AL group, bacteriological concentration in drainage fluid could diagnose AL before infection indexes such as CRP, WBC, and PCT.

**Table 2:** Mean values of bacteriological concentration and pH in drainage fluid between the two groups.

	Bacteriological concentration (/uL)	pH
Non-AL group	356 ± 510	7.94 ± 0.78
AL group	8813 ± 8765	7.72 ± 0.76
*P* value	< 0.001	0.769

**Table 3:** Sensitivity and specificity of bacteriological concentration in pelvic drainage used as a cut-off in the identification of AL (ROC analysis).

Bacteriological concentration (/uL)	Sensitivity (%)	Specificity (%)
923	100	91.04
937.5	100	91.40
964.5	100	91.76
985.5	100	92.11
1034	100	92.47
1096.5	100	92.83
1143.5	100	93.19
1254.5	90.48	93.19
1339	90.48	93.55
1352	90.48	93.91
1385.5	90.48	94.27
1423.5	71.43	94.27
1451.5	71.43	94.62
1501.5	71.43	94.98
1546	71.43	97.49
1680	71.43	97.85

### pH results in drainage fluid

We summarized the lowest pH value of drainage fluid of each patient and compared between the two groups. The results showed that there was no difference between the two groups (*p* = 0.769) (Table 2).

## Discussion

Up to now, surgical operation is the main treatment of rectal cancers. AL is one of the most serious complications of rectal resection, resulting in sepsis, increasing the risk of local and distant metastasis, and reducing overall in-hospital survival^8,9^. Therefore, prediction and early diagnosis of AL following rectal surgery is very important. A series of studies have investigated the risk factors of AL. According to the literature, there are more than thirty preoperative factors are screened, such as male sex, diabetes, low level site of the tumor, calcium scores, Charlson Comorbidity Index (CCI) score, stage IV rectal cancer, liver cirrhosis/severe fibrosis, neoadjuvant chemoradiotherapy, perioperative bleeding, K-ras mutation, anemia, ASA class, chronic obstructive pulmonary disease, smoking history, weight loss, previously infected wound, omitting mechanical bowel preparation, preoperative oral antibiotic use , wounds classified as contaminated or dirty/infected, elderly status, Body Mass Index (BMI), preoperative serum total proteins, use of corticosteroid and so on^10–13^. Besides, there are a lot of intra-operative risk factors are screened such as a high volume of blood loss, endoscopic mucosal grade, side-to-end/J pouch-to-end anastomosis, intramucosal pH, high inferior mesenteric artery ligation (above left colic artery), intra-operative complications, multiple firings of the linear stapler, length of surgery > 3 h, number of units of transfused blood, height of the colonic doughnut < 4.5 mm, low tissue oxygen saturation, and poor blood supply^14–16^. According to these factors, we could predict AL before or during the operation and take some measures to prevent AL.

Early diagnosis of AL is necessary and important when the anastomosis has completed. However, the early diagnosis is usually difficult as the lack of clinical manifestation. When patients present with fever, infection or abdominal symptoms, the diagnosis of AL maybe deferred as these symptoms usually occur when the infection of abdominal cavity is very severe^17^. Therefore, a series of studies have investigated potential biomarkers to predict AL early. Among these, CRP, WBC, PCT, and cytokines in serum were hot study biomarkers, which were proved to be reliable predictors of AL. However, owing to the limit of sensitivity and specificity, we should explore new ideal biomarkers for early diagnosis of AL.

In our opinion, biomarkers in drainage fluid may be potential to detect AL accurately and early according to the definition of AL. As when AL happens, exception occurs firstly in anastomoses, therefore, testing the drain around the anastomoses may get the sign early. Further, when AL happens, the intestinal juice may flow into the abdominal cavity. The intestinal juice contains a lot of bacteria and is alkaline (pH 8.3–8.4), so we presume that testing the concentration of bacteria and the pH of drain may contribute to detecting AL. There were few studies focusing on this topic. Ruiter et al. performed a study and showed that there was different composition of the microbial flora in the peritoneal drainage fluid according to the location of the perforation. The most frequently predominant anaerobes were Bacteroides and isolated aerobic organisms were *E. coli*, Klebsiella, and Pseudomonas species and the predominant anaerobes were Bacteroides in lower gastrointestinal perforation^18^. According to the result above, Elyamani et al. performed a prospective study including fifty-six patients with rectal cancer who underwent elective low anterior resection. They collected peritoneal samples from the abdominal drains on the first, third, and fifth days postoperatively for bacteriological study. The result showed that intraperitoneal bacterial colonization was significantly higher in patients with clinical evidence of AL and they concluded that intraperitoneal bacterial colonization might be an additional diagnostic tool that can support the decision making of surgeons for early detection of anastomotic leak in colorectal surgery^7^. Similarly, Komen et al. showed that quantitative PCR for E. faecalis performed on drain fluid may be an objective, affordable and fast screening tool for symptomatic colorectal AL^19^. However, there is an issue of timeliness in this study. The aerobic organisms should be cultivated for 48 h and anaerobes should be cultivated for 96 h. The poor timeliness limits the clinical application of this technology.

In our study, we tested bacteriological concentration in peritoneal drainage fluid instead of the specific bacteria and the result could be got in 20 min. Our results showed that the bacteriological concentration in the drain of AL group was significantly higher than that in non-AL group. The AUC value was 0.98 according to the ROC curve. The best cut-off value was 1143/uL and the sensitivity and specificity were 100% and 93.19% respectively. The concentration of bacteria in the drain not only had a high value in early diagnosis of AL but also got easily and quickly. In addition, we discovered that date when the drainage fluid exceeded the cut-off of bacterial concentration was earlier than the date when clinical or radiological signs were present. As a result, the concentration of bacteria in the drain was a good marker for early diagnosis of AL and should be widely used.

Precious studies revealed that the pathophysiological character of AL is acute inflammation. Martínez et al. showed that neutrophils played an important role in acute inflammatory process and thinking that interstitial acidic pH characterizes most inflammatory microenvironments. Extracellular acidosis enhanced neutrophil activity and extend00ed its functional lifespan and then intensified the acute inflammatory responses^20^. According to this theoretical foundation, the pelvic draining is becoming acidic when AL is developing. Liu et al. performed a study to evaluate the utility of pH of postoperative pelvic drainage in the identification of AL following anterior resection of rectal cancer. They got a conclusion that an early and persistent decline of pH value of pelvic drainage fluid after rectal surgery with anastomosis, is a marker of AL. A cut-off value of 6.978 determined at 25 ℃ on POD3 maximizes sensitivity and specificity^21^. However, in our study, we got no difference of pH value between the AL and non-AL groups. The reason may be that we treat the AL earlier than the peritoneal drainage fluid around the anastomotic stoma becoming acidic. Multi-center clinical studies are needed to confirm this conclusion.

Along with the advance of ERAS, patients who underwent rectal resection may discharge before AL appears. So that early diagnosis of AL is very important and necessary. Up to now, there was no definite biomarker for early diagnosis of AL. According to the definition of AL, when AL happens, exception occurs firstly in anastomoses, so testing the drain around the anastomoses may get the sign early. Therefore, we should focus on the drainage fluid and discover new ideal biomarkers. We think that the bacteriological concentration in the drainage fluid might be a right maker for early diagnosis of AL. In our clinic work, this marker could diagnose AL before clinical symptoms present and before infection indexes elevating. Then we could treat AL in time, preventing AL with class B from developing into AL with class C. Besides, this marker could guide the time of the removal of drainage tube and guide the discharge. With the development of ERAS, many patients discharged within one week after operation, however, AL mainly appeared 5th-7th postoperative day. It took in mean 8.8 days (range 2–42) until the AL was diagnosed^22^, so we could test this marker before pulling out drainage tube. We should not remove drainage tube and discharge the patient if this marker was beyond the normal range. Of course, this study has limitations, firstly, the sample was small and the number of patients present with AL was small too. There was no validation attempt in the study. Therefore, we will perform a large sample study to validate the conclusion.

## Conclusion

According the results of present study, a high bacteriological concentration in peritoneal drainage fluid is a good marker for predicting and diagnosing AL following rectal resection. The best cut-off value is 1143/uL and the sensitivity and specificity are 100% and 93.19% respectively. However, owing to the limitation of the sample, there was no validation attempt in the study. A large sample study is needed to validate the conclusion.

## Abbreviations

AL: Anastomotic leakage
CRP: C-reactive protein
PCT: Procalcitonin
ISREC: International study group of rectal cancer
CCI: Charlson Comorbidity Index
BMI: Body mass index
WBC: White blood cell
